# Simultaneous Analysis of Ursolic Acid and Oleanolic Acid in Guava Leaves Using QuEChERS-Based Extraction Followed by High-Performance Liquid Chromatography

**DOI:** 10.1155/2017/2984562

**Published:** 2017-07-11

**Authors:** Chang Xu, Yiyi Liao, Chunyan Fang, Makoto Tsunoda, Yingxia Zhang, Yanting Song, Shiming Deng

**Affiliations:** ^1^Key Laboratory of Tropical Biological Resources of Ministry of Education and Department of Pharmaceutical Sciences, College of Marine Science, Hainan University, Haikou 570228, China; ^2^Graduate School of Pharmaceutical Sciences, The University of Tokyo, 7-3-1 Hongo, Bunkyo-ku, Tokyo 113-0033, Japan

## Abstract

In this paper, a novel method of QuEChERS-based extraction coupled with high-performance liquid chromatography has been developed for the simultaneous determination of ursolic acid (UA) and oleanolic acid (OA) in guava leaves. The QuEChERS-based extraction parameters, including the amount of added salt, vortex-assisted extraction time, and absorbent amount, and the chromatographic conditions were investigated for the analysis of UA and OA in guava leaves. Under the optimized conditions, the method showed good linearity over a range of 1–320 *μ*g mL^−1^, with correlation coefficients above 0.999. The limits of detection of UA and OA were 0.18 and 0.36 *μ*g mL^−1^, respectively. The intraday and interday precision were below 1.95 and 2.55%, respectively. The accuracies of the UA and OA determinations ranged from 97.4 to 111.4%. The contents of UA and OA in the guava leaf samples were 2.50 and 0.73 mg g^−1^, respectively. These results demonstrate that the developed method is applicable to the simultaneous determination of UA and OA in guava leaves.

## 1. Introduction

**Figure 1:**
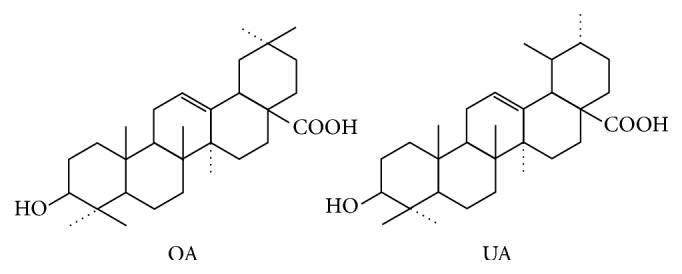
Chemical structures of oleanolic acid (OA) and ursolic acid (UA).

Guava (*Psidium guajava* L.) is a tropical plant that belongs to genus* Psidium* in the Myrtaceae family. Many parts of this plant, especially its fruits and leaves, are utilized by humans. Its fruits are extremely popular because of their flavour and aroma. Reportedly, guava fruits can also be used to treat diabetes mellitus patients and people who have high level blood cholesterol because the fruits contain abundant amino acids, minerals, and vitamins. Meanwhile, guava leaves have been reported to have pharmacological properties, such as hypoglycaemic [1], antimicrobial [2], anti-inflammatory [3], and antinociceptive [4] activities. Ursolic acid (UA) and oleanolic acid (OA) are isomeric triterpenic acids (Figure 1), which are the principal active components in guava leaves. Pharmacological experiments have demonstrated that UA can modulate a variety of signaling pathways associated with cancer survival and progression and has the ability to cure inflammation or oxidative stress-associated diseases (cancer, cardiovascular disease, and neurodegeneration) [5], and OA displays several pharmacological activities, such as anti-inflammatory, antioxidant, anticancer, and hepatoprotective effects [6]. The contents of UA and OA are very low, and their analysis was easily interfered by the complex matrix compounds in guava leaves. Therefore, the establishment of an accurate and easy analytical method for these compounds is particularly important.

For the trace analysis of target analytes, sample preparation, which includes both clean-up and preconcentration before chromatographic analysis, is indispensable for many types of samples. Several sample pretreatment methods such as ultrasound-assisted extraction (UAE) [7], microwave-assisted extraction (MAE) [8], accelerated solvent extraction (ASE) [9], solvent bar microextraction (SBME) [10], and solid-phase extraction (SPE) [11] have been reported for the extraction and enrichment of UA and OA from different matrices prior to analysis by gas chromatography (GC) or liquid chromatography (LC). Among these methods, SPE is increasingly being used as a sample preparation technique because of its advantages of reducing sample handling, labour, and organic solvent consumption. The most popular sorbents for SPE are octadecyl (C_18_)-bonded silica or polymeric sorbents. Other sorbents that enhance selectivity are immunosorbents and molecularly imprinted polymers (MIPs). However, the SPE method often fails to remove interferences when a large number of samples is extracted. Other problems include low recovery, incomplete elution, poor conditioning, and high variability due to method and operator variability. However, the main disadvantage of SPE is its unconfirmed effectiveness at extracting water-soluble metabolites.

Currently, a new sample preparation method, QuEChERS, which stands for “quick, easy, cheap, effective, rugged, and safe,” combined with GC or LC, has been used for the multiclass, multiresidue analysis of pesticides and natural products in fruits, vegetables, and herbs. QuEChERS can serve as a template for modification because of its characteristic flexibility and can be adjusted for various matrices depending on the analyte properties, matrix composition, equipment, and analytical technique. Oshita and Jardim compared QuEChERS-based dispersive and cartridge SPE for the analysis of pesticides in strawberries and found that the QuEChERS-based dispersive SPE extraction clean-up of the strawberry extracts was more efficient, achieving higher pesticide recoveries and better clean-up via interference removal than those reported to date [12]. Tölgyessy et al. have successfully applied the QuEChERS method with Dual dispersive SPE and gas chromatography to the determination of ten chlorinated priority substances in fish [13]. Li et al. proposed an effective method based on QuEChERS to extract triazines and phenylureas from milk and yogurt [14]. Raihanah et al. used the QuEChERS preparation method to analysis imidacloprid residue in paddy samples [15].

The aim of this work was to develop an effective method based on QuEChERS-based extraction coupled with high-performance LC (HPLC) for the simultaneous detection of UA and OA in guava leaves. In this study, QuEChERS Kits were used for the pretreatment of guava leaves. The method utilizes a combination of primary and secondary amines (PSA) to remove organic acids, anhydrous MgSO_4_ to reduce the remaining water, and C_18_ to remove lipids and waxes. Besides, the extraction parameters affecting the sample recoveries were optimized, and the obtained accuracy and precision demonstrated that the proposed method is applicable for the determination of UA and OA in guava leaves.

## 2. Materials and Methods

### 2.1. Materials

Standards of UA and OA (purity is more than 98%) were purchased from Shanghai Yuanye Biotechnology Co., Ltd. (Shanghai, China). Triethylamine and acetate (HPLC grade) were obtained from Aladdin Industrial Corporation (Shanghai, China). Methanol (HPLC grade) was purchased from Fisher Scientific (Beijing, China). Ultrapure water was obtained from a Milli-Q water purification system (Millipore, USA). All the other reagents were analytical grade. Extraction was performed using Agilent SampliQ QuEChERS AOAC Extraction Kits (p/n 5982-5122), Agilent Bond Elut Florisil (12256014), Dikma ProElut C_18_ (500 mg/6 mL), and Dikma ProElut PLS (500 mg/6 mL).

### 2.2. Standard Solutions

Stock solutions of UA and OA (1 mg mL^−1^) were prepared in methanol. Mixed working solutions, containing 0.4 mg mL^−1^ UA and 0.2 mg mL^−1^ OA, were prepared by appropriate dilution of the stock solution with methanol. All solutions were stored at 4°C before use.

### 2.3. Sample Preparation

Guava leaves were collected from the campus of Hainan University and pulverized into powder. Approximately 2.5 g of the powder was accurately weighed, transferred into a clear beaker, dissolved in 15 mL of methanol, and then ultrasounded for 1 h. The solution was transferred to a 25 mL volumetric flask, brought to volume, and filtered through a funnel. The filtrate was subsequently centrifuged (Hermle, Germany) at 13000 rpm for 5 min at 25°C. Finally, the supernatant was collected.

### 2.4. Procedure

The extraction procedure consisted of several steps. First, the centrifuged sample (2 mL) containing the analytes was transferred to a QuEChERS Kit containing 150 mg of MgSO_4_, 50 mg of PSA, and 50 mg of C_18_ EC sorbent. After adjusting the pH to 7 by adding appropriate amounts of 10 mol/L NaOH solutions, the capped tubes were shaken for 3 min by hand and then vortexed for 1 min on a mechanical shaker to facilitate the dispersion of the sample with 3%* w/v *salt addition. Finally, the capped tubes were centrifuged at 8000 rpm for 5 min at 4°C. The supernatant was filtered and then evaporated until near dryness with gentle nitrogen stream at 40°C. Finally, the residue was dissolved in 2 mL methanol, filtered through a 0.2 *μ*m nylon syringe filter (JinTeng, China), and 5 *μ*L of filtrate was injected into the HPLC.

Agilent Bond Elut Florisil column was activated with acetone/n-hexane (5 mL, 1 : 9, v/v) and conditioned with n-hexane (5 mL) before application; after the sample had passed through the column, 2 mL of the methanol extractants was loaded on the cartridge and then eluted with acetone/n-hexane (5 mL, 1 : 9, v/v). The eluent was collected and then evaporated until near dryness with gentle nitrogen stream at 40°C [16]. Finally, the residue was dissolved in 2 mL methanol, filtered through a 0.2 *μ*m nylon syringe filter (JinTeng, China), and 5 *μ*L of filtrate was injected into the HPLC.

Dikma ProElut C_18_ and Dikma ProElut PLS column were activated with 5 mL of methanol and 5 mL of Milli-Q water before use, respectively. Two microlitres of the methanol extracts passed through the column and then were washed with 5 mL of Milli-Q water and 5 mL of phosphate buffer (0.05 M, pH = 8.5), the eluent discarded. The analyte was then eluted with 5 mL of methanol; the eluent was collected and then evaporated until near dryness with gentle nitrogen stream at 40°C [17]. Finally, the residue was dissolved in 2 mL methanol, filtered through a 0.2 *μ*m nylon syringe filter (JinTeng, China), and 5 *μ*L of filtrate was injected into the HPLC.

### 2.5. Chromatographic Conditions

HPLC analysis of UA and OA was performed on an HPLC instrument as described above. The mobile phase consisted of methanol-water-acetate-triethylamine (90 : 10 : 0.04 : 0.02, v/v/v/v). The flow rate was 1.0 mL min^−1^, with UV detection at 210 nm. The column temperature was maintained at 30°C. Five microlitres of the centrifuged supernatant was injected into an HPLC system equipped with a Waters 1525 pump and 2489 detector (Milford, USA) and a Silversil C_18_ analytical column (250 mm × 4.6 mm ID, 5 *μ*m) from Dikma Technologies Inc. (Beijing, China). These were performed based on previous report [18].

### 2.6. Optimization of QuEChERS Procedure

The QuEChERS-based extraction parameters, including the added salt amount, extraction time, and absorbent amount, were investigated. The experiments were all carried out in triplicate using the standard solutions. The results were then checked in guava leaf extract.

### 2.7. Validation Study

A series of standard solutions containing UA and OA (1, 10, 20, 40, 80, 160, and 320 *μ*g mL^−1^) were prepared for the establishment of the calibration curve. The intraday precision was determined by analyzing the QuEChERS-based extracted guava leave samples five times in the same day. The interday precision experiment was performed by analyzing the QuEChERS-based extracted guava leave samples once a day for five consecutive days. The reproducibility was evaluated by six QuEChERS-based extracted samples in the completely same procedure. The recovery of the method was assessed by the addition of standard solutions with different concentrations into the extracted sample obtained in Section 2.3. The recovery (*R*) was calculated based on the following equation:

(1) R=analytesample with spike−analytesample without spikeanalyteadded×100%.

## 3. Results and Discussion

### 3.1. Effect of the Amount of Salt Addition

**Figure 2:**
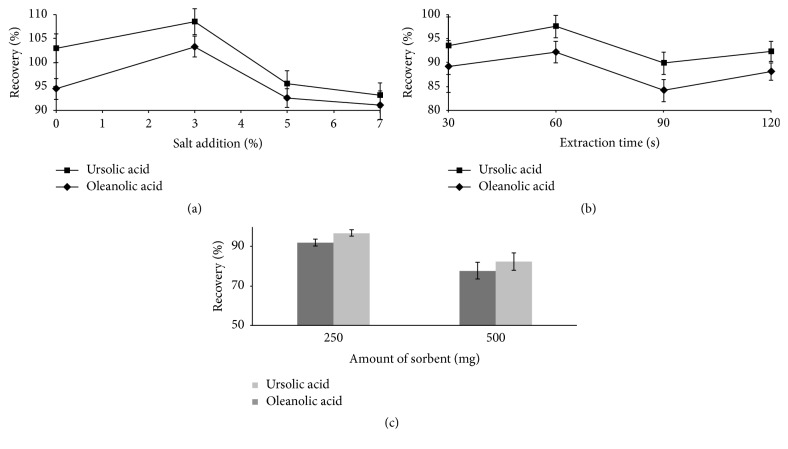
Effect of salt addition (a), extraction time (b), and sorbent amount (c) on the QuEChERS-based extraction of standard solutions.

Salt was added directly to partition the organic and aqueous layers through a higher salting-out effect. A concentration of 0–7% (*w/v*) sodium chloride was added to the standard solution to examine the effect of the amount of salt addition on the extraction efficiency. The peak areas of the analytes increased when the NaCl concentration was increased from 0–3% (*w/v*) and decreased when the NaCl concentration was further increased (Figure 2(a)). The addition of salt into solution can significantly reduce the solubility of polar substances; however, when the salt concentration is too high, the viscosity of the solution will increase, resulting in reduced adsorbent extraction capacity. Therefore, 3% (*w/v*) salt addition was utilized in further studies.

### 3.2. Effect of the Extraction Time

The extraction time is particularly important for the mass transfer process, and the vortex extraction time was varied from 30 to 120 s. The extraction efficiency increased when the extraction time was extended from 30 to 60 s and slightly decreased when the extraction time was extended to 120 s (Figure 2(b)). This decrease may have been due to the back extraction of analytes from the sorbent into the sample solution. Therefore, 60 s was used in subsequent analyses as the optimum extraction time.

### 3.3. Effect of the Sorbent Amount

The effect of the sorbent amount on the extraction efficiency was investigated at 250 mg and 500 mg of sorbent. A total of 250 mg of sorbent consisted of 150 mg of MgSO_4_, 50 mg of PSA, and 50 mg of C_18_ EC, and 500 mg of sorbent contained the same components in double the quantity. The results presented in Figure 2(c) indicate that the maximum recovery was achieved by using 250 mg of sorbent to extract 2 mL of sample solution. Hence, 250 mg of sorbent was used in the subsequent experiments.

### 3.4. Optimum QuEChERS-Based Extraction Conditions of Guava Leaf Samples

Additional experiments were performed to check the optimum QuEChERS-based extraction conditions of guava leaf samples. The results showed that the optimum QuEChERS-based extraction conditions are consistent with what we did in standard solutions with the presence of other matrix analytes.

### 3.5. Method Validation

Under the optimum conditions, the proposed method was validated in terms of the linearity, limit of detection (LOD), limit of quantification (LOQ), precision, and recovery.

#### 3.5.1. Linearity, Limit of Detection, and Limit of Quantification

Good linearity was observed for UA and OA, with correlation coefficients *r*^2^ of 0.9997 and 0.9991, respectively. The LODs and LOQs, based on signal-to-noise ratios of 3 (*S*/*N* = 3) and 10 (*S*/*N* = 10), were found to be in the range of 0.18–0.36 and 0.6–1.2 *μ*g mL^−1^, respectively. These results illustrate the lower detection limits of UA and OA of the proposed method. All of the analytical characteristics are summarized in Table 1.

#### 3.5.2. Intraday and Interday Precision

To evaluate the precision of the method, five similar experiments were carried out with samples on the same day and on five consecutive days. The intraday relative standard deviation (RSD%) ranged from 1.48 to 1.95% and the interday precision ranged from 1.18 to 2.55% (Table 1). The relative standard deviation (RSD) for the peak area is 2.52%. These results show the good reproducibility and precision of this method.

#### 3.5.3. Recovery

A recovery study was carried out by spiking methanol extracts in Section 2.3 with three different concentrations of UA (60, 120, and 180 *μ*g mL^−1^) and OA (12, 24, and 36 *μ*g mL^−1^). The results summarized in Table 1 show that the recoveries of UA and OA were within a range of 97.4 to 111.4%, and, in RSD, they ranged from 0.38 to 7.14%. UA and OA are planar molecules and QuEChERS sorbents are not efficient to retain planar molecules [19]; therefore, good recoveries were achieved.

### 3.6. Analysis of Guava Leaf Samples

**Figure 3:**
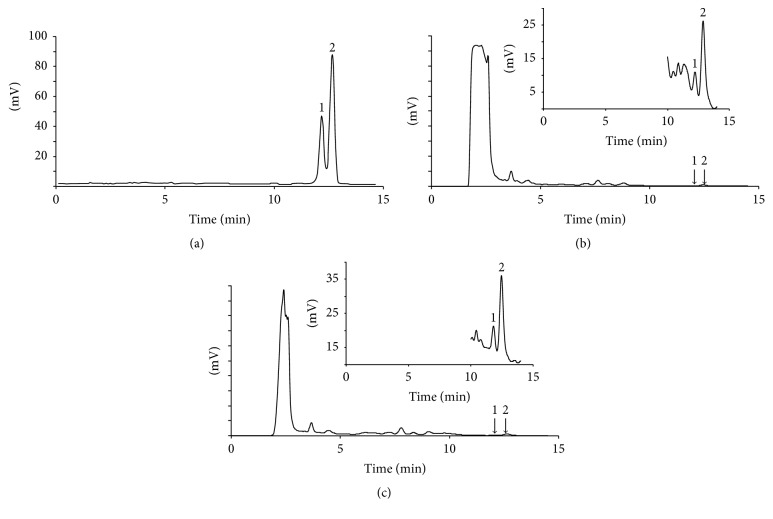
HPLC chromatograms of standard solutions of UA and OA (a), a blank guava leaf sample (b), and a guava leaf sample after QuEChERS-based extraction (c). The embedded graphs are the magnified view of the chromatograms. Peaks in the chromatogram: (1) OA, (2) UA.

The developed method was applied to the determination of UA and OA in guava leaf samples. The contents of UA and OA in the guava leaf samples were 2.50 and 0.73 mg g^−1^, respectively. These values were in good agreement with those reported in previous study [20].  Figure 3 shows HPLC chromatograms of standard solutions of UA and OA (a), a blank guava leaf sample (b), and a guava leaf sample after QuEChERS-based extraction (c).

### 3.7. Comparison with Other Reported Methods

**Figure 4:**
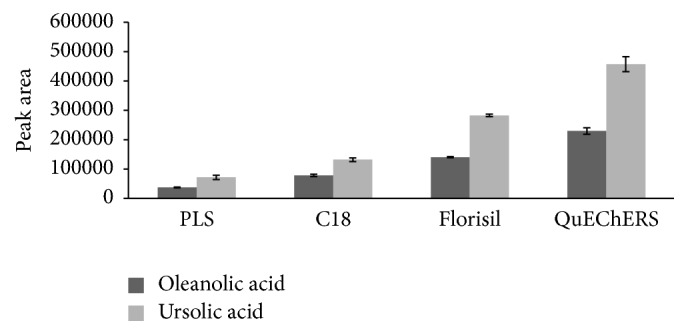
Peak area of the guava leave samples in different extraction columns.

QuEChERS method was compared with other three solid-phase columns, including Agilent Bond Elut Florisil column, Dikma ProElut C_18_ column, and Dikma ProElut PLS column.  Figure 4 presented that the peak areas of UA and OA obtained by QuEChERS method were obviously higher than the other three columns and indicated that QuEChERS method achieved higher recovery of UA and OA in guava leaves comparing with other three extraction methods. Moreover, the proposed method yields higher recoveries and reproducible results. Overall, QuEChERS-based extraction combined with HPLC-UV is a useful alternative approach for the preconcentration and determination of UA and OA in complex matrices.

## 4. Conclusions

In this study, a method consisting of QuEChERS-based extraction combined with HPLC was developed and optimized to quantitatively determine trace levels of UA and OA in guava leaves. The extraction and clean-up procedures are simple and time-saving. Moreover, compared with other solid-phase extraction methods, the proposed method yields higher recoveries of UA and OA in guava leaves. The validated method provides good precision, a wide linear range, and low LOD and LOQ in the *μ*g mL^−1^ range for the analysis of guava leaf samples. The results suggest that QuEChERS-based extraction combined with HPLC-UV can be a useful alternative approach for the preconcentration and determination of UA and OA in complex matrices.

## Figures and Tables

**Table 1:** Validation data for the target analytes in guava leaves.

Analyte	Concentration range (*μ*g mL^−1^)	Calibration curve	LOD (*μ*g mL^−1^)	LOQ (*μ*g mL^−1^)	Spiked level (*μ*g mL^−1^)	Precision, RSD%		Relative recovery, % (RSD, %) (*n* = 5)
						Intraday (*n* = 5)	Interday (*n* = 5)	
Oleanolic acid	1–160	*y* = 2611*x* − 909.59	0.36	1.2	12	1.95	2.55	109.0 (0.38)
					24			97.4 (3.32)
					36			108.2 (3.18)
Ursolic acid	2–320	*y* = 1950*x* + 6240.8	0.18	0.6	60	1.48	1.18	101.6 (4.10)
					120			99.2 (2.53)
					180			111.4 (7.14)
